# Time efficient whole-brain coverage with MR Fingerprinting using slice-interleaved echo-planar-imaging

**DOI:** 10.1038/s41598-018-24920-z

**Published:** 2018-04-27

**Authors:** Benedikt Rieger, Mehmet Akçakaya, José C. Pariente, Sara Llufriu, Eloy Martinez-Heras, Sebastian Weingärtner, Lothar R. Schad

**Affiliations:** 1 Computer Assisted Clinical Medicine, University Medical Center Mannheim, Heidelberg University, Mannheim, Germany; 2 Magnetic Resonance Image Core Facility, Institut d’Investigacions Biomèdiques August Pi i Sunyer (IDIBAPS), Barcelona, Spain; 3 Center of Neuroimmunology. Laboratory of Advanced Imaging in Neuroimmunological Diseases, Hospital Clinic Barcelona and Institut d’Investigacions Biomediques August Pi i Sunyer (IDIBAPS), Barcelona, Spain; 4 Electrical and Computer Engineering, University of Minnesota, Minneapolis, MN United States; 5 Center for Magnetic Resonance Research, University of Minnesota, Minneapolis, MN United States

## Abstract

Magnetic resonance fingerprinting (MRF) is a promising method for fast simultaneous quantification of multiple tissue parameters. The objective of this study is to improve the coverage of MRF based on echo-planar imaging (MRF-EPI) by using a slice-interleaved acquisition scheme. For this, the MRF-EPI is modified to acquire several slices in a randomized interleaved manner, increasing the effective repetition time of the spoiled gradient echo readout acquisition in each slice. Per-slice matching of the signal-trace to a precomputed dictionary allows the generation of T_1_ and T_2_* maps with integrated B_1_^+^ correction. Subsequent compensation for the coil sensitivity profile and normalization to the cerebrospinal fluid additionally allows for quantitative proton density (PD) mapping. Numerical simulations are performed to optimize the number of interleaved slices. Quantification accuracy is validated in phantom scans and feasibility is demonstrated *in-vivo*. Numerical simulations suggest the acquisition of four slices as a trade-off between quantification precision and scan-time. Phantom results indicate good agreement with reference measurements (Difference T_1_: −2.4 ± 1.1%, T_2_*: −0.5 ± 2.5%, PD: −0.5 ± 7.2%). *In-vivo* whole-brain coverage of T_1_, T_2_* and PD with 32 slices was acquired within 3:36 minutes, resulting in parameter maps of high visual quality and comparable performance with single-slice MRF-EPI at 4-fold scan-time reduction.

## Introduction

Quantification of tissue properties has long been an overarching goal in Magnetic Resonance Imaging (MRI) research, allowing for inter-patient and inter-scan comparability^1^. Recently, signal quantification has achieved major clinical impact in multiple fields of MRI^2–6^. Neurological applications of quantitative MRI have gained interest with the introduction of magnetic resonance fingerprinting (MRF)^7^, due to the premise of fast simultaneous multi-parameter quantification. MRF is based on generating unique signal signatures, termed “fingerprints,” for different tissue types based on their underlying MRI properties. This is achieved by the rapid acquisition of numerous images with varying contrast weightings induced by the variation of sequence parameters including flip angle and echo time (TE). Matching these fingerprints to a precomputed dictionary allows parameter mapping of relaxation parameters including T_1_, T_2_ and T_2_* ^8,9^, tissue properties such as perfusion^10^ and system parameters such as B_1_^+ ^^11^. MRF has been used in clinical studies to evaluate the range and progression of MRF-derived relaxometry values in the brain as a function of the age of healthy volunteers^12^. Recently, a study demonstrated that MRF can differentiate common types of adult brain tumors, providing initial evidence for its clinical utility^13^.

The original MRF method was based on a balanced steady state free precession^7^ sequence design with highly undersampled spiral readout allowing for joint T_1_ and T_2_ mapping. Unbalanced fast imaging with steady state precession (FISP)^8,14–16^ was subsequently introduced to overcome sensitivity to B_0_ field inhomogeneities at the expense of reduced signal-to-noise ratio (SNR). Recently, we introduced an alternative MRF sequence for simultaneous T_1_ and T_2_* mapping based on spoiled gradient echo imaging with Cartesian echo-planar imaging k-space readout (MRF-EPI)^9^, potentially fostering robustness towards gradient deviations and trajectory inaccuracies compared with unspoiled spiral readouts^17^.

While MRF has successfully enabled efficient multi-parameter quantification in a single-slice, its applicability with improved coverage has been limited. Conventionally, full magnetization relaxation needs to be ensured prior to the acquisition of the next slice in order to use the same signal model for each measurement. A recent study has proposed shortened relaxation intervals, maintaining similar quantification accuracy at the cost of compromised precision due to lower SNR in the baseline images^18^. Further, simultaneous multi-slice (SMS) imaging^19^ has been incorporated into MRF^20,21^ by creating time-varying phase modulation between the acquired slices, in order to alleviate the problem of confined coverage and to increase scan-time efficiency. While obtaining an acceleration factor up to 3, computationally complex kernel fitting is needed for complete slice separation and additional training data must be acquired prior to the measurement, increasing overall measurement time. Further, quantification precision is compromised due to overlapping coil-geometries in the SMS reconstruction^20^. Most recently, 3D MRF methods were also proposed for improved spatial coverage^22,23^. In these studies highly regularized image reconstructions^23^ or repeated acquisition from the steady-state^22^ were used to enable reconstruct of a continuous imaging volume from a 3D stack-of-spirals k-space.

Slice-interleaved acquisition is a complimentary approach for volumetric imaging and clinical standard in numerous applications^24^, including diffusion MRI, fMRI and gradient echo sequences. Slice-interleaved schemes achieve similar SNR compared to 3D^25^ acquisitions, while allowing arbitrary slice spacing. Compared to single-slice imaging scan-efficiency is substantially improved due to increased effective TR, leading to higher SNR for each slice. However, the need for coherent signal-paths limits the effective TR in balanced sequence designs^26^, so far preventing the use of slice-interleaved acquisitions in MRF.

In this study, we sought to increase scan-time efficiency of volumetric coverage in MRF parameter mapping by integrating a slice-interleaved acquisition scheme in MRF-EPI. Spoiled gradient echo readouts enable increased effective TRs, ultimately enabling whole-brain coverage in clinically acceptable scan-times. The number of interleaved slices is numerically optimized to provide a trade-off between scan-time and quantification precision. Phantom experiments are performed to validate quantification accuracy of joint T_1_, T_2_* and proton density (PD) mapping. *In-vivo* images in healthy subjects and patients suffering from multiple sclerosis are obtained in order to study feasibility of whole-brain quantification for clinical usage and compare image quality to single-slice acquisitions.

## Materials and Methods

### Pulse sequence design

**Figure 1:**
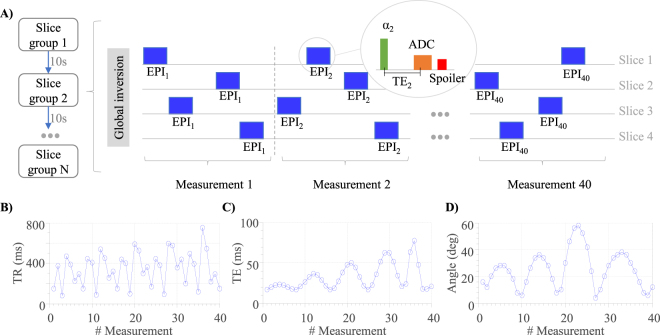
(**A**) Schematic diagram of the slice-interleaved MRF-EPI comprising multiple consecutive slice groups, each acquiring four slices with multiple EPI readouts; slice order is randomized within each measurement. Profiles of the repetition time (**B**) echo time (**C**) and flip angle variations (**D**) used for the proposed MRF measurements.

MRF-EPI^9^ is modified to allow for slice-interleaved acquisition of multiple slices (Fig. 1A). Following a global inversion pulse, numerous single-slice EPI readout modules are acquired in rapid succession. The slice position is varied in a pseudo-random fashion. The randomization is performed block-wise for groups containing the acquisition of each slice once. Slice order within these groups is randomly permuted. This ensures that each slice is sufficiently sampled throughout the measurement, while creating pseudo-random signal traces. Especially in the initial stages of the acquisition this guarantees that the sampling frequency of each slice is similar during the early part of the inversion recovery, ensuring comparable sensitivity. The pseudo-randomization influences the effective slice-TR (Fig. 1B), which includes the acquisition time of the other slices, leading to higher signal dissimilarities of the resulting fingerprints. As shown in Fig. 1C,D, TE and flip-angles are also varied to obtain sensitive tissue fingerprints as previously proposed^8^. Fat-suppression was included to minimize EPI imaging artifacts; gradient spoiling was incorporated by using crusher gradients of equal polarity performed before and after the fat-suppression pulse. For whole-brain coverage multiple interleaved slice-group acquisitions were performed, each simultaneously measuring four slices (Fig. 1A), separated by a 10 second pause to guarantee full magnetization recovery due to the global inversion pulse.

### Dictionary

The dictionary was generated off-line using MATLAB (The MathWorks; Natick, MA) by simulating the evolution of the magnetization based on Bloch-equation simulations on a per-slice basis, as detailed by Rieger *et al*.^9^. B_1_^+^ compensation was integrated by simulating a scaling factor to the flip-angle excitation pulse^14^. Dictionary matching was performed by choosing the entry with the highest inner product between the magnitude of the dictionary entry and the magnitude of the measured signal. Due to the varying TR pattern of each slice within a slice group, a separate dictionary was precomputed for each unique TR pattern. Each per-slice MRF dictionary consisted of 157,938 entries with the following parameter range: B_1_^+^ amplitude correction^14^ using a linear scaling factor in the range of 0.6–1.4 in steps of 0.1, T_1_ = 20–2000 ms in steps of 10 ms, and 2000–6000ms in steps of 500 ms; T_2_* = 10–100 ms in steps of 2 ms, and 100–300 ms in steps of 5 ms. Unrealistic entries with T_1_ < T_2_* were discarded.

### Proton Density (PD) Mapping

Numerous factors influence the voxel intensity in an MRI measurement, and need to be accounted for when quantifying the tissue PD^1^. In the present sequence, relaxation induced signal changes are incorporated as T_1_ and T_2_* in the dictionary model. Transmit radiofrequency field B_1_^+^ inhomogeneities are compensated using scaling of the effective flip-angle as described above. Semi-quantitative *M*_0_ maps, can be calculated from the matching dictionary entry *f* and the measured signal *k*, using a least-square fit with the closed form solution of

1 $${M}_{0}=\frac{|{k}^{T}k|}{|{k}^{T}f|}$$

Here, the semi-quantitative *M*_0_ is characterized as

2 $${M}_{0}=C\cdot \rho \cdot S,$$

depending on PD *ρ*, coil sensitivities *S* and a constant scaling factor *C* that includes spatial-invariant scaling such as receiver gain and DICOM export window-leveling. The coil sensitivity maps *S* were obtained with the method described by Volz *et al*., based on the idea, that the proton density map is a combination of the data set M_0_ with full spatial resolution and a bias field comprising of low spatial frequencies^27^. It was shown that the coil sensitivity S can be calculated with a probabilistic framework including optimised parameters^28^, by which *in-vivo* images are registered, segmented and bias-corrected. The model incorporates a smoothness intensity variation estimation termed field bias, which is proportional to *S*^27^. The field bias map was calculated from the *M*_0_ map using the segmentation toolbox of the SPM12 software package (http://www.fil.ion.ucl.ac.uk/spm), using the default toolbox parameters: Regularization = 0.001, FWHM = 60 mm cutoff. To quantify *ρ*, *C* is calculated using cerebrospinal fluid (CSF) as a reference point, such that 100 percentage units (pu) corresponds to the known PD of 110.3 mol/l at 37° ^29^. The scaling factor was determined by manually placing regions of interests (ROIs) in CSF and normalizing these areas to 100pu.

### Numerical Simulations

For a given scan-time the proposed sequence requires a trade-off between number of slices per slice group and number of baseline images per slice. However, an increased number of slices also increases the average effective slice TR, leading to higher baseline SNR. To determine the optimal number of slices for a given measurement time of 17 seconds per slice group, the stability of the parameter quantification from the MRF data was evaluated in dependence of the number of baseline images by estimating the noise amplification in the linearized MRF system for T_1_ and T_2_* parameters as described by Rieger *et al*.^9^. The average normalized noise amplification was calculated for fingerprints generated from 18 pairs of relaxation times in the *in-vivo* range (T_1_: 1000–2500 ms T_2_*: 50–70 ms) for 1 to 160 baseline images, each. The TE and flip angle patterns were interpolated according to the number of measurements and TR was maximized to reach a measurement time of 17 seconds.

To verify the analysis based on linearization, Monte-Carlo simulations were performed to determine the quantification accuracy. The same set of fingerprints, TE, TR and flip angles patterns were used as in the noise amplification simulation. Noise was added (n = 1000) to the simulated patterns and matched with the dictionary. The mean normalized quantification accuracy was calculated depending on the number of baseline images.

The randomization of the slice order was performed to achieve more homogenous quantification characterization across the slices, as compared with sequential order. Numerical simulations comparing the quantification precision between these slice ordering schemes are provided in Supplementary Information.

### Acquisition

To test the performance of the sequence, phantom and *in-vivo* data were acquired on a 3T whole-body scanner (Magnetom Trio; Siemens Healthcare, Erlangen, Germany) using a standard 32-channel head array coil for *in-vivo* measurements and 30-channels of a body and spine array for phantom scans. This study was approved by the local institutional review board (Institutional Review Board II, Medical Faculty Mannheim, Germany), all subjects provided written informed consent prior to examination and all methods were performed in accordance with the relevant guidelines and regulations. For the proposed slice-interleaved MRF acquisition, following parameters were used: 4 slices per slice group, TE/TR = 17–78 ms/80–755 ms, flip angle = 4–58°, FOV = 220 × 220 × 140 mm^3^, slice-gap = 0.9 mm, voxel size = 1.0 × 1.0 × 3.0 mm^3^, band-width = 1136 Hz/pixel, partial-Fourier = 5/8, parallel imaging factor 3 with GRAPPA reconstruction, reference lines = 48 acquired in-place for each baseline image, acquisition time per slice group = 17 s, total number of baseline images = 160 (4 slices with 40 images each). The single-slice MRF-EPI used the same parameters, though only acquiring one slice with 160 baseline images and an acquisition time per slice = 17 s.

### Phantom Experiments

The accuracy and precision of the sequence were evaluated in phantom experiments and compared to reference measurements, inversion-recovery turbo spin-echo for T_1_ (IR-TSE, 6 images, TI = 50–4000 ms, TE/TR = 6 ms/15 s, turbo factor = 16, matrix/FOV = 64 × 128/110 × 220 mm^2^, slice thickness = 3 mm, BW = 399 Hz/pixel, scan-time = 4 min 30 sec, two-parameter fit) and spoiled gradient echo for T_2_* (GRE, 6 images, TE = 5–300 ms, TR = 1000 ms, alpha = 15°, matrix/FOV = 64 × 128/110 × 220 mm^2^, slice thickness = 3 mm, BW = 390 Hz/pixel, scan-time = 6 min 18 sec, three parameter fit). Each phantom was acquired 10 times in separate measurements with the slice-interleaved MRF-EPI and single-slice MRF-EPI. Reference measurements were acquired once per phantom. The nine phantoms consisted of a single gadoterate-meglumine (Dotarem; Guerbet, Villepinte, France, concentration: 0.075–0.15 µmol/ml) doped agarose compartments each (concentration: 0.5–1.5%). The mean relaxation times were determined by manually drawing ROIs in the phantoms. The accuracy of the slice-interleaved MRF-EPI was determined by comparing the average deviation and the normalized root-mean-square error (NRMSE) between the method and the reference measurements. A two sample Student’s t-test was used to conclude if the single-slice and slice-interleaved MRF-EPI have significantly different means. P values less than 0.05 were considered to be significant.

Consistency within a slice group was tested to study quantification differences among the slices caused by different acquisition parameters. For each phantom four separate measurements (A, B, C, D) were performed with the slice-interleaved MRF sequence, each acquiring four slices (A1, …, A4, and B1, …B4, …). The slice group location was shifted among the four measurements in such a way that the center of the phantom was covered by a different one of the four slices in each measurement (i.e. the center of the phantom was covered by A1, B2, C3 and D4). Consistency among the four slices was defined as the difference between the measurement in A1, B2, C3 and D4, using the same manually drawn ROI.

PD mapping was evaluated in a phantom consisting of gadoterate-meglumine doped water. Reference PD maps were acquired using multiple GRE images with long TR to avoid T_1_ weighting, and varying echo-time to compensate for T_2_* decay (GRE, 5 images, TE = 3–60 ms, TR = 1500 ms, alpha = 90°, matrix/FOV = 64 × 64/220 × 220 mm^2^, BW = 390 Hz/pixel, scan-time = 7 min 50 sec, three parameter fit). Imaging was performed with the body coil, which was used both for transmit and receive. B_1_^+^ maps were acquired using a double-angle method (GRE, 2 images, TE = 10 ms, TR = 1500 ms, alpha = 45°/90°). Given the reciprocity assumption, as transmit and receive were performed with the same body coil, B_1_^+^ maps were also used for receive coil profile correction. Accordingly, PD maps were calculated with $\rho =C\cdot I\cdot {B}_{1}^{+}\cdot \,S/{e}^{-TE/{T}_{2}\ast }$. The reference and slice-interleaved measurements were performed with a slice thickness of 10 mm with varying amounts of water and air within the slice, thus changing the PD depending on the water-air ratio. PD values of the slice-interleaved EPI and the reference measurements were compared by placing ROIs within the PD maps and acquiring average values in each. The constant scaling factor was chosen such that 100% water is normalized to 100pu in manually drawn ROIs in a full water compartment. The B_1_^+^ maps acquired using a double-angle method were also used in the correction of the MRF-EPI PD instead of bias field correction, as the latter probabilistic method is known to be unsuitable for phantom measurements^28^.

### *In-vivo* experiments

Whole-brain *in-vivo* MRF quantifications were acquired with the proposed method in 6 healthy volunteers (4 men, 31 ± 6 years old) and 4 multiple sclerosis (MS) patients (2 men, 42 ± 5 years old). T_1_, T_2_* and PD values were obtained for white matter and grey matter by segmenting a slice of each healthy volunteer with the segmentation toolbox of SPM12. To avoid partial voluming effects in small structures, the masks were eroded with MATLAB image erosion algorithm (disk radius = 1 pixel). T_1_, T_2_* and PD for MS patients were determined by manually placing ROIs in the lesions, as identified on separate clinical measurements.

### Data availability statement

The datasets generated during and the current study are not publicly available as data clean up and further analysis is currently underway but are available from the corresponding author on reasonable request.

## Results

### Numerical Simulations

**Figure 2:**
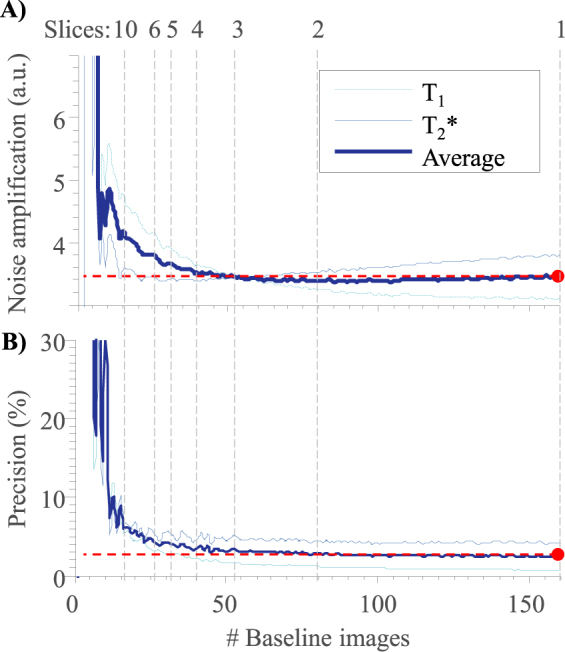
(**A**) Average normalized noise amplification and (**B**) dictionary matching precision of a Monte-Carlo simulation for a range of T_1_ (1000–2500 ms) and T_2_* (50–70 ms) values in dependence of the number of baseline images. Both graphs show only minor variations between 40–160 baseline images, corresponding to 1–4 slices.

Figure 2A shows the average noise amplification in the parameter recovery from MRF data as a function of the number of baseline images. The combined average noise amplification of T_1_ and T_2_* (thick blue curve) has a similar amplitude for 40 to 160 baseline images. The noise amplification increases rapidly, when reducing the number of images below 40. The separate T_1_ and T_2_* curves have different characteristics, while the T_2_* noise amplification is lowest with 29 baseline images, it increases slightly with more images, implying that for T_2_* quantification fewer higher SNR baseline images have higher noise resilience than many low SNR images. T_1_ has lowest noise amplification with a high number of baseline images with a slight increase until 20 baseline images. For less than 20 images, the amplification is rapidly rising, as a too low number of baseline images causes the inversion recovery curve to be sampled sparsely, decreasing noise resilience. Figure 2B shows the normalized quantification precision in the Monte-Carlo simulations as a function of the number of baseline images. The results show the same characteristics as the noise amplification using the linearized system (Fig. 2A) with similar average precision for 40 to 160 baseline images. Reducing the number of baseline images increases the effective TR, thereby enabling the acquisition of other slices during these pauses. As the average noise amplification has a similar value for the baseline images between 40 and 160, acquiring 4 slices for each slice group in the proposed acquisition results in the highest acceleration factor with only marginal loss of precision.

### Phantom Experiments

**Figure 3:**
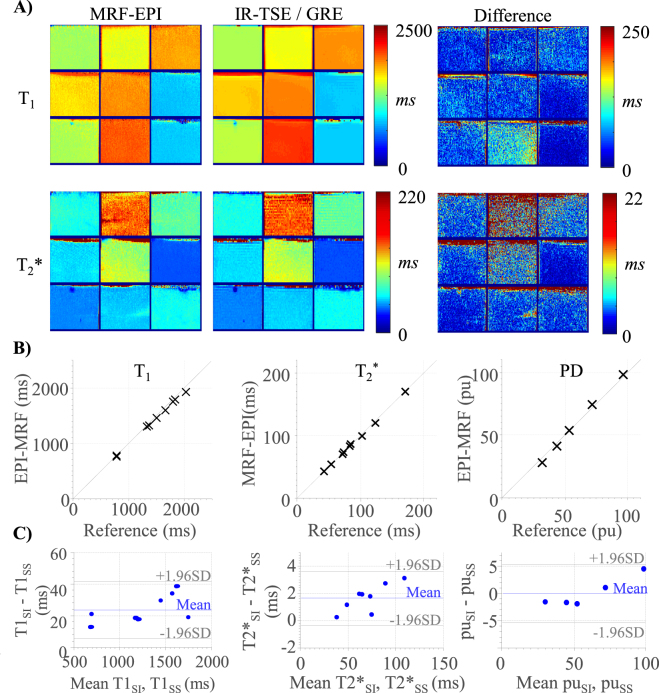
(**A**) T_1_ and T_2_* maps obtained in phantom measurements using slice-interleaved MRF-EPI and the respective reference method IR-TSE and GRE. (**B**) Comparison of T_1_, T_2_* and proton density (PD) values of slice-interleaved MRF-EPI with the reference methods showing nearly identical average quantification in all phantoms. (**C**) Bland-Altman plot showing good agreement between the slice-interleaved MRF-EPI (T1_SI_, T2*_SI_) and the single-slice MRF-EPI (T1_SS_, T2*_SS_).

Figure 3A shows the T_1_ and T_2_* maps of the phantom experiments using MRF-EPI and reference measurements. The proposed method yields homogeneous T_1_ estimates within the phantoms. T_2_* maps show a higher degree of variation than T_1_ maps, due to magnetic susceptibilities and large field inhomogeneities influencing the T_2_* values, both in the reference and MRF measurements. Figure 3B depicts the T_1_, T_2_* and PD quantification using the slice-interleaved MRF-EPI as compared to the reference method. Proposed MRF-EPI shows slight underestimation of T_1_ (deviation: −2.4 ± 1.1% [min: −4.5%, max −0.8%], NRMSE: 3.0%), T_2_* (−0.5 ± 1.5% [min: −2.7%, max 1.6%], NRMSE: 1.5%) and PD (−0.5 ± 7.2 pu [min: −11.6 pu, max 6.3 pu], NRMSE: 6.5%) values compared to the reference measurements.

The Bland-Altman plots in Fig. 3C compare the quantification accuracy of the single-slice MRF-EPI to the slice-interleaved MRF-EPI for T_1_ and T_2_*. No significant difference was found between the single and slice-interleaved MRF (p ≤ 0.05). The slice-interleaved implementation results on average in slightly higher values for T_1_ (24 ± 9 ms [min: 12 ms, max: 38 ms])) and T_2_* (2 ± 1 ms [min: 0 ms, max 3 ms]) maps.

Precision and accuracy were not affected by different TR patterns among the 4 slices within a slice group, resulting in a deviation of less than 1% for all parameters when quantifying the exact same slice location in a phantom as a test for inter-slice consistency.

### *In-vivo* Experiments

**Figure 4:**
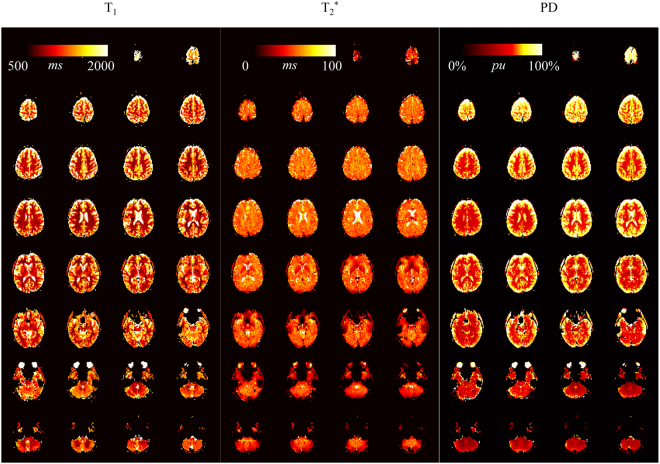
T_1_, T_2_* and proton density maps acquired with slice-interleaved MRF-EPI, 32 slices with a resolution of 1 × 1 × 3 mm were measured within a total measurement time of 3:36 minutes.

**Figure 5:**
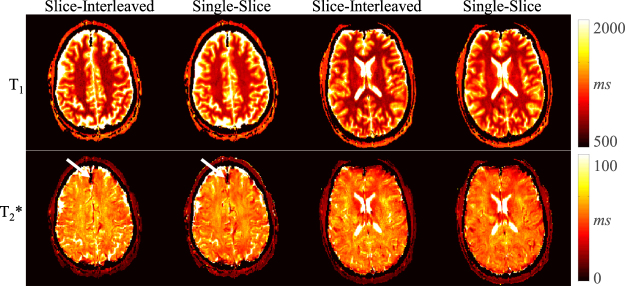
Exemplary *in vivo* T_1_ and T_2_* maps acquired in one subject with slice-interleaved and single-slice MRF-EPI. Both techniques achieve visually comparable image quality, with good white/grey matter delineation in the T_1_ maps and susceptibility contrast weigthing in the T_2_* maps. Intracranial calcification is clearly depicted by signal dropout in the T_2_* map of both sequences (white arrow).

**Figure 6:**
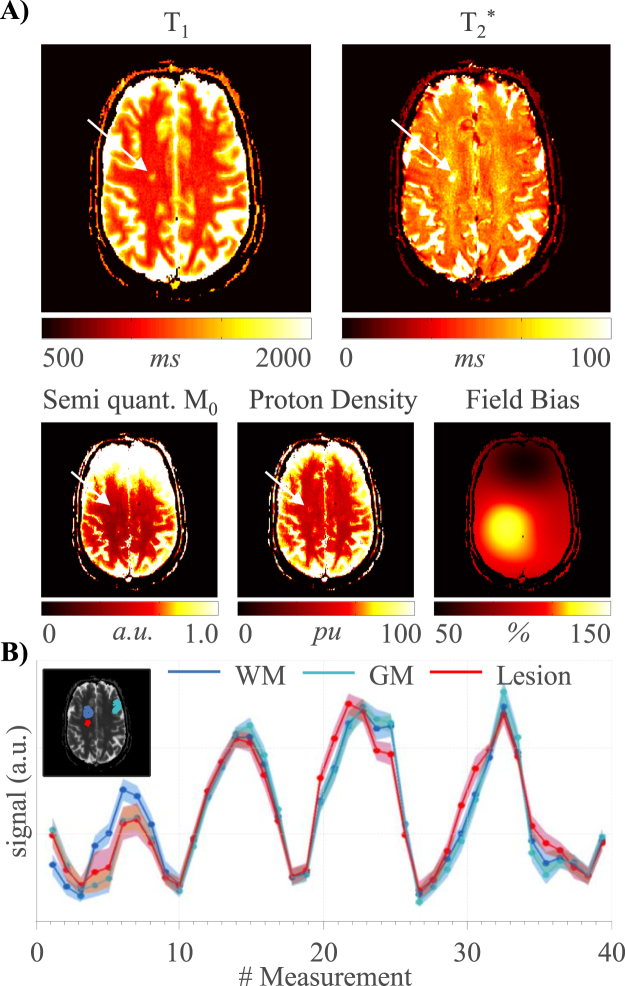
(**A**) T_1_, T_2_*, semi quantitative M_0_, corrected proton density and field bias map of an MS patient with clearly visible lesions (white arrow), (**B**) Exemplary fingerprints from manually drawn ROIs in grey matter (GM), white matter (WM) and a lesion.

Full brain MRF data with 32 slices was successfully acquired in all healthy volunteers and patients. Figure 4 shows representative T_1_, T_2_* and PD maps from a healthy volunteer. The mean T_1_/T_2_*/PD values of all healthy volunteers of an exemplary slice are: white matter: 746 ± 57 ms/57 ± 6 ms/72 ± 7 pu, grey matter 1200 ± 100 ms/53 ± 6 ms/92 ± 18 pu. While T_2_ values are generally higher in grey than white matter, similar values for T_2_* in grey and white matter are obtained. This is well in line with previous studies and attributed mostly to magnetic susceptibility^30^. Figure 5 shows two example slices of a healthy volunteer acquired with the slice-interleaved and single-slice MRF-EPI. Both methods yield visually comparable T_1_ and T_2_* maps. The volunteer has intracranial calcifications in the frontal lobe of the brain (white arrow), leading to signal dropouts in the region in T_1_ as well as T_2_* maps, which is visible in both methods. Results from a patient scan are depicted in Fig. 6 (female, 40 years). The lesion of the MS-patient is clearly visible in T_1_, T_2_* and PD maps (Fig. 6a, further images in Supplementary Figure S2). Exemplary T_1_, T_2_* and PD values of lesions from three MS patients are higher compared to surrounding tissue (1285 ± 200 ms/95 ± 17 ms/62 ± 0 pu, 1528 ± 113 ms/121 ± 21 ms/62 ± 0 pu, 1270 ± 66 ms/81 ± 12 ms/60 ± 0 pu), allowing clear quantitative discrimination especially in the T_2_* maps. Sample fingerprints of the MS patient retrieved from manually placed ROIs in grey matter, white matter and a lesion display clearly differentiable signal paths (Fig. 6B). The same trend and good differentiability is observed across all subjects, despite minor subject specific variations.

## Discussion

In this work, we integrated slice-interleaved scanning in the MRF-EPI method to increase scan-time efficiency, enabling quantification with increased volumetric coverage with an acceleration factor of 4 compared to single-slice MRF. Acquisition of slice groups with four slices provided high quantification accuracy in phantom experiments, comparable to the single-slice implementation and in agreement with reference measurements. Whole-brain *in-vivo* scans with 32 slices were acquired within 3:36 minutes in multiple volunteers, resulting in robust and artifact-free T_1_, T_2_* and PD parameter maps.

In comparison to the original MRF-EPI with 160 baseline images, a reduced number of 40 baseline images were acquired per slice. Our numerical simulations of noise amplification and phantom experiments showed that a reduced number of baseline images with longer TR and therefore higher SNR can lead to comparable quantification precision compared with a high number of low SNR baseline images. The results indicate that by reducing the number of baseline images while increasing TR, T_2_* accuracy is enhanced due to the higher SNR images. T_1_ accuracy declines slightly, as less data is acquired during the inversion recovery period at the start of the measurement. Our simulations indicate that acquiring four interleaved slices sharing a global inversion pulse leads to the highest scan-time efficiency gain for values in the *in-vivo* range, without compromising accuracy. The T_1_ precision could possibly be improved by including further inversion pulses during the acquisition of the slices, facilitating even higher acceleration. Alternative sequence designs incorporating this idea warrant investigation in future studies.

Simultaneous multi-slice imaging has recently received increasing interest^19^, including quantitative applications^31,32^, as it provides means for scan-time acceleration where the only loss in SNR is due to coil geometries. SMS has recently been applied to trueFISP-based MRF in two studies^20,33^. While gaining a 3-fold acceleration, higher factors are currently limited by noise amplification and signal leaking. Further, the sequence needs to acquire additional training data per slice and the computationally intensive regridding algorithms pose a challenge, while the quantification precision is compromised. Combining SMS with EPI by applying a cyclic phase-shift among the k-space lines allows for an intuitive interpretation of the CAIPIRINHA approach as FOV shifts induced in separate bands. SMS-EPI is commercially available from a major vendor and is successfully integrated into several clinical and large scale cohort studies with previously reported acceleration rate of up to 16^34^. Therefore, the combination of SMS with the proposed slice-interleaved scheme bears great promise for ultra-rapid whole-brain quantification of multiple parameters.

Several methods have been recently proposed to shorten reconstruction times by altering the process of dictionary matching^35–37^. These can be readily incorporated in the quantification step of the proposed method, replacing conventional dictionary matching, in order to speed up post-processing. However, due to a lower number of baseline images, reconstruction times are typically less of a concern, as compared with other MRF methods. In the proposed scheme, as well as in other MRF techniques, spatial resolution is limited by the readout duration per image. Advanced undersampling and reconstruction or denoising techniques have been recently proposed in the context of MRF^37–44^ to facilitate increased undersampling rates or to improve noise performance, by exploiting structure or inter-dependencies mainly along the temporal dimension. As these approaches are applicable to a variety of sampling schemes or have specifically been demonstrated for EPI^45^, they are fully compatible with the proposed slice-interleaved MRF method. Due to the increased baseline SNR in the proposed approach, these reconstruction methods promise large gains in the feasible undersampling factor at minor loss in quantification quality. This may ultimately facilitate high-resolution quantification without scan-time penalty and warrants investigation in future studies.

Volumetric MRF sequences based on highly undersampled stack of spirals trajectories have demonstrated the feasibility of whole-brain quantitative T_1_ and T_2_ imaging. Liao *et al*.^22^ minimized scan time by including Cartesian GRAPPA with a factor of 3 in k_z_ direction and simultaneously reducing the baseline images to 420 per slice, compared to 1000 in the original MRF-FISP sequence. However, to allow for sufficient k-space data to reconstruct a 3D volume per imaging contrast, repeated acquisitions of the signal train are performed from a steady-state. This enabled whole-brain quantification with 1 mm isotropic resolution within 7.5 min, although with extensive reconstruction complexity amounting to ~20 h computation time. Ma *et al*.^23^ used an interleaved sampling pattern, acquiring 4 interleaved slices per group and sequentially measuring multiple groups, while also reducing the number of baseline images per slice to 480 to decrease scan time. Further, the relaxation time between the slice groups was set to 3 seconds to reduce scan time, therefore preventing full relaxation before the acquisition of the next slice group, as previously proposed^18^. Within 5 min T_1_ and T_2_ maps of 48 slices with a resolution of 1.2 × 1.2 × 3.0 mm were acquired, though an additional B_1_ measurement was needed to correct for B_1_ inhomogeneity effects and improve the accuracy of T_1_ and T_2_ estimates. To achieve reconstruction of an imaging volume without the need for repeated acquisitions per contrast, image regularization was integrated with a sliding window reconstruction across multiple images. Several recent works have enabled 3D EPI or echo volumar imaging (EVI), based on highly, accelerated imaging Cartesian readouts^46,47^. Readout-times could be further shortened by exploiting dependencies between the acquisition of multiple images^46^, or by employing image regularization^48^. These methods offer interesting potential to enable the acquisition of multiple interleaved 3D volumes in the proposed sequence scheme, similar to the method of Ma *et al*. The combination of slice-interleaving and volumetric coverage allows to synergistically benefit of the SNR gain of both methods and will be subject of future studies.

Slice-interleaved 2D and 3D acquisitions are complimentary techniques for volumetric coverage, each offering a distinct profile of advantages. The SNR gain is reported to be very similar in many practical applications^25^. Comparing the proposed slice-interleaved MRF-EPI to a hypothetical 3D acquisition with varying coverage but constant scan time, the 3D acquisition would achieve constant SNR, due to $SNR \sim {d}_{x}{d}_{y}{d}_{z}\sqrt{{T}_{acq}}$ ^49^. For 1 to 4 slices, no drawback in terms of SNR is observed for the slice-interleaved implementation as shown by the simulations (Fig. 2). Hence, in this regime the slice-interleaved MRF-EPI provides comparable to SNR to an idealized 3D implementation. While 3D sequences commonly allow for lower minimal slice thickness and improved slice profiles, they often result in higher undersampling of the k-space and might require more elaborate reconstructions schemes, while conventional reconstructions are applicable to the proposed scheme. Further, 3D sequences are limited to the acquisition of a continuous volume. Multislice 2D allow for arbitrary slice spacing, enabling time efficient coverage of larger volumes without compromising resolution, by using slice gaps. Thus slice-interleaved acquisitions are often preferred in clinical applications, including scout and overview scans, which have the highest demand in terms of volumetric coverage.

In the present sequence, relaxation periods are required between different slice-groups due to the application of a non-selective adiabatic inversion-pulse. This can be circumvented by the application of slice-selective inversion. However, this requires a multi-band inversion pulse covering all spatially separated slices within the slice group and introduces substantial contrast weighting on the B_1_^+^ profile of the inversion-pulse. B_1_^+^ correction of the inversion similar to the one in proposed by Buonincontri *et al*.^14^ can be integrated, and warrants further studies. Recently, it was shown that the relaxation periods can be significantly shortened between MRF pulse train repetitions by starting the repetition using a non-relaxed initial spin state^18^. While quantification accuracy is maintained, increased computational complexity is needed as the shortened relaxation times must be accounted for within the Bloch simulations. This approach might be used in combination with slice-interleaved MRF in future studies to minimize wait times and reduce acquisition time, at the cost of reduced precision due to lower baseline SNR.

Integrating B_1_^+^ compensation in MRF has been shown to improve quantification precision. This has been done by either using a Bloch-Siegert reference scan prior to the MRF measurement^50^ or by integration a scaling factor to the flip-angle excitation pulse in the dictionary simulation. As previously evaluated in MRF-EPI, the latter method was chosen as to increase quantification accuracy in the presence of imperfect excitation slice profiles and inhomogeneities in the transmit field without the need for additional scan time. However, this scheme assumes a single flip-angle representative of the slice-profile. This has been shown to be a valid approximation for small flip-angles in steady-state conditions^51^. To further overcome residual inaccuracies which might be observed with high flip-angles or pseudo-random acquisitions, actual slice-profile simulations can also be integrated in the dictionary, albeit at increased computational complexity.

The present slice-interleaved implementation has a similar computational complexity for the dictionary matching process to the single-slice MRF-EPI. On the one hand, four dictionaries need to be calculated, as each slice in a slice group has a unique TR pattern, while on the other hand the dictionary size per slice is reduced. Including slice-interleaved acquisitions in MRF favors a non-balanced spoiled sequence design, as the acquisition of interleaved slices hereby does not affect the signal paths of the other slices. MRF methods based on a balanced sequence design require coherent signal-paths limiting the effective TR, prohibiting the application in slice-interleaving without major adaptation.

PD mapping has a number of clinical applications, including multiple sclerosis^52^ and brain tumors^53^. However, PD mapping is challenging, as traditionally a number of separate measurements need to be performed to compensate for contrast induced intensity variation. These variations include relaxation processes, inhomogeneous transmit and receive fields, as well as potentially other contrast mechanisms^1^. MRF has been proposed as a promising method for fast, joint quantification of a number of parameters. Previous studies have included a first-step towards MRF PD mapping by providing semi-quantitative M_0_ maps^7^. However, to achieve PD quantification additional bias correction for B_1_^+^ and coil sensitivity maps is required. Furthermore, in a balanced sequence design as previously proposed the M_0_ measurement is confounded by residual contrast sensitivity towards molecular diffusion^54^ and magnetization-transfer^55^, potentially necessitating further corrections to obtain reliable PD maps. Due to the spoiled gradient echo contrast in MRF-EPI all necessary bias corrections can be performed in a two-step process without the need of additional measurements. A B_1_^+^ correction scheme was suggested by Buonincontri *et al*.^14^, including flip angle correction in the dictionary matching process, which has been integrated in MRF-EPI^9^. Coil sensitivity correction can be performed by calculating the field bias maps^27^ based on a probabilistic per-image framework^28^. The bias field maps have been shown to have a high *in-vivo* accuracy compared to separately measured coil sensitivity maps^56^. As spoiled gradient-echo imaging is commonly not associated to other contrast sensitivities, the proposed approach compensates for the portfolio of confounders commonly considered in previous PD mapping studies^27^. However, residual inaccuracies can be induced by deviations from the assumed signal model, including non-monoexponential transverse signal decay, as previously reported for complex tissue structures such as lung alveolus^57^.

Patient motion is one of the main causes of artefacts in clinical MRI. While MRF has been shown to be partially resilient to certain kinds of patient movement, initial results demonstrate that generic motion can induce significant intra-image variance to the MRF signal trace, resulting in motion artefacts in parameter maps^58–60^. This effect can be exacerbated if increased scan time is necessary for volumetric coverage. In the proposed scheme, the total duration between first and last data acquisition for any given slice, is comparable to previously proposed single slice sequences. Hence, the sensitivity to patient motion can be expected to be comparable to other MRF techniques. Furthermore, MRF seems well suited for correction of residual in-plane motion using co-registration of the baseline data, due to the rapid image acquisition. Motion correction schemes that are insensitive to contrast variation among the baseline images have been previously proposed^61,62^. Hence, further mitigation of in-plane motion effects using contrast invariant motion correction of the baseline images prior to dictionary matching warrants future investigation.

While the proposed method is used for joint T_1_ and T_2_* quantification, it could be extended for T_1_ and T_2_ quantification by incorporating refocusing pulses prior to the readout. This is commonly performed in multiple applications of EPI^63^, benefiting from increased SNR at the expense of increased minimal TE and longer scan times. However, this would also allow for an integrated assessment of diffusion biomarkers by including randomized diffusion gradients. Extending the portfolio of simultaneous quantification of biomarkers with the proposed method is subject of further research, and may facilitate the usage of these methods for a wider range of diseases.

The study and the proposed method have limitations regarding comparability with other methods and the number of patients. The proposed method uses the same TE and flip angle scheme as the single-slice MRF-EPI method, subsampled by a factor of four. To further increase accuracy or accelerate the sequence, optimization of sequence parameters may be necessary, which will be subject of further research. Due to the lack of the original MRF sequence at our center, no direct comparison could be performed regarding accuracy and precision. Further, in this study only a small number of subjects were scanned to prove the *in-vivo* feasibility. Larger cohorts with specific diseases remain to be evaluated.

## Conclusion

In the study, we have demonstrated the feasibility to accelerate the MRF-EPI for volumetric coverage by a factor of four while maintaining quantification accuracy using a slice-interleaved acquisition scheme. Within 17 seconds four slices with a resolution of 1 × 1 × 3 mm^3^ are acquired, resulting in artifact free T_1_, T_2_* and PD maps.

## Electronic supplementary material

Supplementary Info
