# Supplementary material for: Time efficient whole-brain coverage with MR Fingerprinting using slice-interleaved echo-planar-imaging
Source: Sci Rep. 2018 Apr 27;8:6667. doi: 10.1038/s41598-018-24920-z (PMC5923901; doi:10.1038/s41598-018-24920-z)
Supplement: Supplementary file 1 — Supplementary Info [file 41598_2018_24920_MOESM1_ESM.pdf]

Time efficient whole-brain coverage with MR Fingerprinting using slice-interleaved echo-planar-imaging

B Rieger[1,2], M. Akçakaya, [4,5], J. Pariente[2] S. Llufriu[3], E. Martinez-Heras[3], S.Weingärtner[1,4,5,†,*] and L. R. Schad[1,†]

[1]Computer Assisted Clinical Medicine, University Medical Center Mannheim, Heidelberg University, Mannheim, Germany

[2]Magnetic Resonance Image Core Facility, Institut d'Investigacions Biomèdiques August Pi i Sunyer (IDIBAPS), Barcelona, Spain

[3]Center of Neuroimmunology. Laboratory of Advanced Imaging in Neuroimmunological Diseases. Hospital Clinic Barcelona and Institut d'Investigacions Biomediques August Pi i Sunyer (IDIBAPS)

[4]Electrical and Computer Engineering, University of Minnesota, Minneapolis, MN, United States

[5]Center for Magnetic Resonance Research, University of Minnesota, Minneapolis, MN, United States

†(denotes joint last-authorship)

Corresponding author: Sebastian Weingärtner, sebastian.weingaertner@medma.uni-heidelberg.de

Materials and Methods

The quantification precision of the randomized slice acquisition scheme was compared against a sequential acquisition scheme. For this purpose, a variety of noisy fingerprints (T_1=100-6000ms, T_2*=10-100ms) were simulated based on the Bloch-equations on a per-slice basis with SNR=90 (compared to thermal equilibrium). T_1 and T_2* were quantified from the noisy fingerprints using dictionary matching. Monte-Carlo simulation (n=1000 iterations) were used to measure quantification precision, defined as the standard deviation of the quantified value across the different iterations for each slice separately. The fingerprints were simulated with following parameters: TE =17-78ms, TR=80-755ms, flip angle=4-58°. While for the sequential scheme the TR was constant within each slice-group, the randomized sequence scheme yields TR variation as shown in Figure 1b. The mean and standard-deviation across the four slices was compared between the two slice schemes.

Results

The quantification accuracy of the randomized and sequential acquisition schemes were virtually identical (Randomization / Sequential: T_1 0.07±1.77%/0.07±1.76% T_2*: 1.37±5.35%/1.37±5.39% mean±std across slices). However, quantification precision was more homogenous across slices with the randomized acquisition scheme resulting in lower standard-deviation across the slices compared with the sequential acquisition scheme (Randomization / Sequential: T_1 1.77±**0.09**%/1.76±**0.14**% T_2*: 5.35±**0.13**%/5.39±**0.23**% mean±std across slices) This indicates, that randomizing the slice order during acquisition leads to improved consistency of the quantification precision within a slice group.

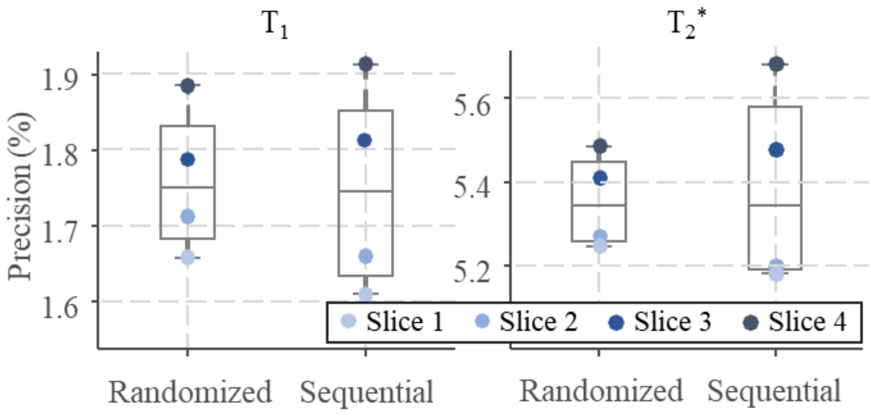

Supplementary Figure S1: Quantification precision of a Monte-Carlo simulation for a range of fingerprints of the randomized and sequential slice shift scheme. Improved homogeneity of the precision across the slices, as represented by smaller standard deviation, is achieved with the randomized scheme.

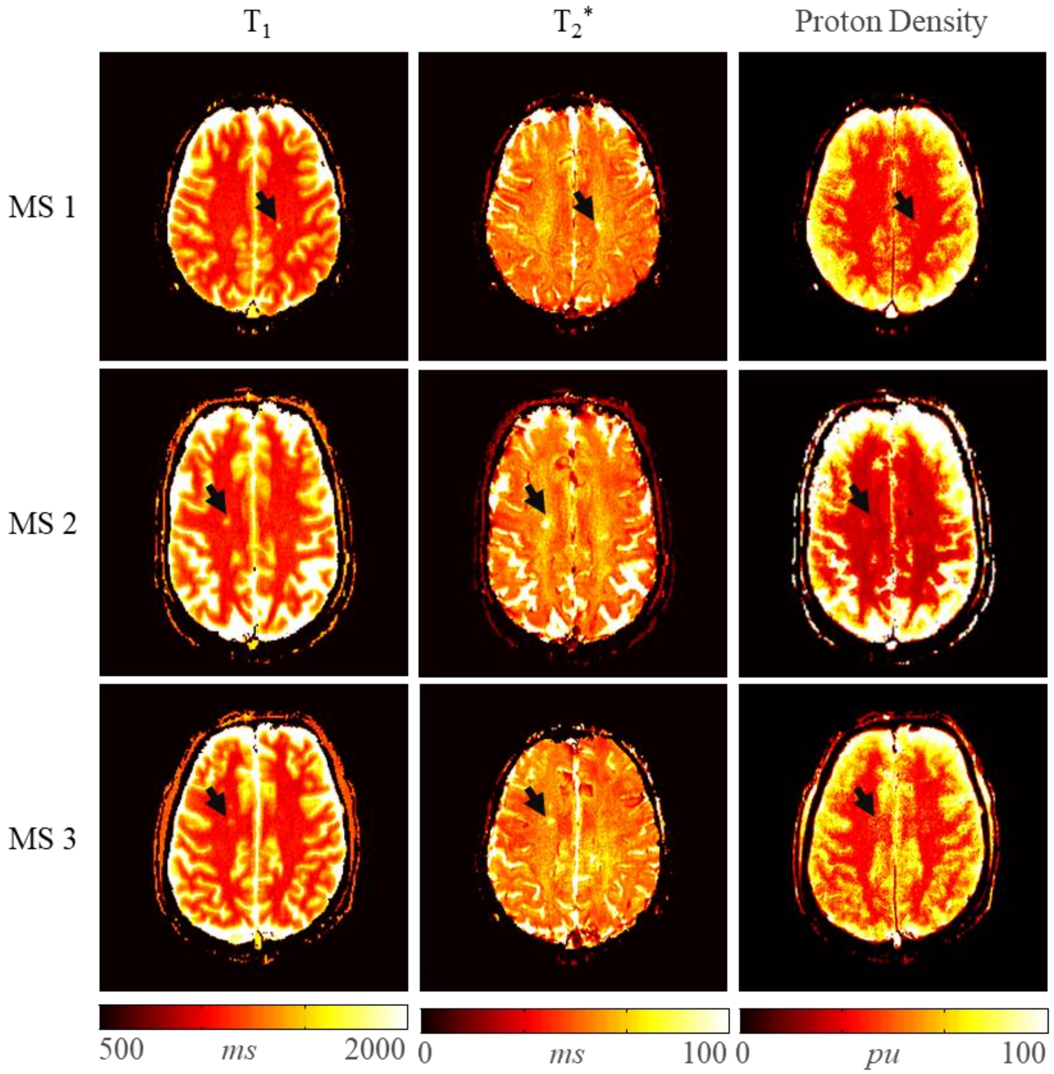

Supplementary Figure S2: T_1, T_2 and corrected proton density map of three MS patients with clearly visible lesions (black arrows)*
